# Neurologic symptoms following COVID-19 in Lima, Peru: a prospective longitudinal observational study

**DOI:** 10.3389/fneur.2025.1524613

**Published:** 2025-07-21

**Authors:** Hanalise V. Huff, Carla Villanueva-Colina, Monica M. Diaz, Sofia Tovar, Andrea Davila Luna, Tianxia Wu, Davidson H. Hamer, Igor J. Koralnik, Tom Solomon, Miguela A. Caniza, Patricia J. Garcia

**Affiliations:** ^1^Department of Global Health and Population, Harvard T. H. Chan School of Public Health, Boston, MA, United States; ^2^Section of Infections of the Nervous System, National Institute of Neurological Disorders and Stroke, National Institutes of Health, Bethesda, MD, United States; ^3^School of Public Health, Cayetano Heredia University, Lima, Peru; ^4^Department of Neurology, University of North Carolina at Chapel Hill School of Medicine, Chapel Hill, NC, United States; ^5^Clinical Trials Unit, National Institute of Neurological Disorders and Stroke, National Institutes of Health, Bethesda, MD, United States; ^6^Department of Global Health, Boston University School of Public Health, Boston, MA, United States; ^7^Section of Infectious Diseases, Boston University Chobanian and Avedisian School of Medicine, Boston, MA, United States; ^8^Boston University Center on Emerging Infectious Diseases, Boston, MA, United States; ^9^Ken and Ruth Davee Department of Neurology, Northwestern University Feinberg School of Medicine, Chicago, IL, United States; ^10^The Pandemic Institute and The National Institute for Health and Care Research (NIHR) Health Protection Research Unit in Emerging and Zoonotic Infections, University of Liverpool, Liverpool, United Kingdom; ^11^The Walton Centre, NHS Foundation Trust, Liverpool, United Kingdom; ^12^Department of Global Pediatric Medicine and Infectious Diseases, St. Jude Children’s Research Hospital, Memphis, TN, United States

**Keywords:** Long-COVID, neurologic, global health, Latin America, COVID-19, Peru, neuroinfectious disease

## Abstract

**Introduction:**

There is limited research on long-term neurologic symptoms following SARS-CoV-2 infection in Peru. This study aimed to describe the longitudinal experience of survivors of mild to moderate COVID-19 in Lima, Peru.

**Methods:**

This prospective, longitudinal observational study included neurologic follow-up data between 3- and 12-months following SARS-CoV-2 infection. Recruitment to a parent study: “Natural History of SARS-CoV-2 in Comparison to Influenza Virus: A Multi-site Study Focused on the Southern Hemisphere and Equatorial Regions” (COFLU Peru), occurred between February 2021 and February 2022 in a Callao, Peru public hospital emergency department. In-person visits for this sub-study, “Neuro COFLU,” included neurologic history and symptom questionnaire.

**Results:**

Fifty-four patients were seen for at least one visit, two of whom required hospitalization for COVID-19. Forty-one (76%) reported at least one pre-existing neurologic diagnosis (59% headaches; 24% migraines). At follow-up visits, patients reported at least one new neurologic symptom since COVID-19: 24/29 (83%) at 90 days, 31/42 (74%) at 180 days, 41/46 (89%) at 270 days, and 20/21 (95%) at 365 days. The median number of new symptoms was 3 at 90 days, 3 at 180, 4 at 270 and 3 at 365 days. Days 90–180 frequent symptoms included: muscular pain, neck stiffness, headache, loss of appetite, numbness, insomnia, and weakness (24–31%). Days 181–365 frequent symptoms included: fatigue, weakness, memory problems, irritability, changes in hearing, muscular pain, joint pain, and insomnia (28–33%). Pre-existing anxiety was associated with post-COVID-19 hearing changes, muscular pain, numbness and weakness and pre-existing depression with neck stiffness and numbness. No significant association was found with age, sex, vaccination status, or pre-existing headaches. Full recovery was reported for 6/29 (21%) at day 90, 13/42 (31%) at day 180, 17/46 (37%) at day 270, and 9/20 (45%) at day 365. By day 365, patients reported median recovery of 85–90%.

**Discussion:**

Persistent neurological symptoms are common in COVID-19 survivors in Lima. Many new neurologic symptoms persisted and increased in prevalence over 3–12 months. Limitations include lack of control group and small sample size. Longitudinal studies of outcomes are needed to predict and mitigate the long-term physical, social and economic effects of SARS-CoV-2 infection.

## Introduction

The coronavirus disease 2019 (COVID-19) pandemic devastated many individuals, health care systems and economies worldwide with often the greatest impact on low-resource settings. Though the COVID-19 mortality rate and impact on health systems differed based on country, it became clear that, irrespective of borders, survivors of the acute infection were reporting a constellation of persistent symptoms across the globe (1, 2).

“Long-COVID” or Post-Acute Sequalae of COVID-19 (PASC) are both terms used to refer to the persistent symptoms following the acute infectious phase of COVID-19 which often include fatigue, “brain fog,” sleep disturbances, and shortness of breath (3). A WHO-led Delphi process including countries representing all World Bank income groups, as well as patients and caretakers as stakeholders, compiled a consensus definition. “Post-COVID-19 condition” was defined as a set of symptoms experienced by adults with a history of probable or confirmed SARS-CoV-2 infection usually >3 months prior and lasting at least 2 months. These symptoms cannot be explained by an alternative diagnosis, they generally have an impact on everyday functioning, they may be either new after recovery or persistent from initial illness, and they may also fluctuate or relapse over time (4). The National Academies of Sciences released a similar, but slightly different, definition of Long-COVID and acknowledged that definitions may be dynamic as more knowledge is gained about the complications following this novel virus (5) and what constitutes Long-COVID may ultimately differ based on context.

The nervous system is known to be affected during the acute phase of COVID-19 (6), with one early report out of Wuhan, China showing that 36% of hospitalized cases had neurologic symptoms (7). A later study looking at neurology consults on hospitalized COVID-19 patients in a US safety-net hospital found most frequent neurologic symptoms to include: altered mental status, fatigue and headache (8). Other reports have described anosmia in mild disease and encephalopathy in severe disease (9, 10). One meta-analysis found stroke affected 2% of all patients with acute COVID-19 (11). Given common Long-COVID symptoms reported early on, the nervous system has also been implicated in the post-acute phase following COVID-19.

Peru, a culturally diverse low-to-middle income country in Latin America, with one of the highest inequality indices in the world, had the highest mortality due to COVID-19 with 89.4 deaths per one hundred thousand (12–14). It has been stated that the country saw a crumbling of the health care system with a peak in deaths in April 2021 (15) as the pandemic unfolded with scarcity of hospital beds and medical resources, such as oxygen (16). In the first months of the pandemic, an estimated 17.8% of deaths occurred outside the hospital including at homes, shelter, on public roads or in transit to health care facilities (14) while the in-hospital mortality rate from COVID-19 found in one large academic hospital during the first wave of the pandemic was 41.3% (17). The older adult population of Peru experienced the most profound effect with an estimated death excess of 139.7% in the first 6 months of the pandemic compared to the same time frame of previous years (18). By March 2023, there were an estimated 4.5 million cases and 220,000 deaths in Peru (15) with case fatality rate of about 4.9% (19).

At the time of this study’s initiation, there was relatively limited information about clinical presentation of COVID in Peru, and even less about the clinical course after the acute phase of illness. Additionally, there are few reports looking at the specific neurologic challenges of Peruvian patients following COVID-19. This study aims to describe the longitudinal experience of survivors of mild to moderate COVID-19 in Lima, Peru with an emphasis on neurologic symptoms reported. Our objective was to determine the prevalence and characteristics of neurologic symptoms following recovery from acute COVID-19 in an urban Peruvian population.

## Materials and methods

### Study population

This study, “Neuro COFLU,” was a prospective, longitudinal observational study which included neurologic follow-up data from patients that had been recruited for a larger study entitled “Natural History of SARS-CoV-2 in Comparison to Influenza Virus: A Multi-site Study Focused on the Southern Hemisphere and Equatorial Regions (COFLU Peru).” COFLU Peru recruited individuals 18 years or older who met the clinical case definition for COVID-19 at time of enrollment with estimated symptom duration of 96 h or less and provided informed consent for participation. The recruitment happened at a hospital emergency department in the Callao region of Peru between February 2021 and February 2022. All patients had SARS-CoV-2 PCR performed in the emergency department. Only those with a positive PCR test remained in the study for follow up at different intervals for up to 365 days after enrollment.

There were 1,467 participants who underwent initial evaluation for COFLU Peru, of whom 468 met eligibility criteria and 303 agreed to participate. Two-hundred ninety-five provided informed consent and 94 met inclusion criteria for COFLU Peru with 86 with SARS-CoV-2 positive PCR test. We enrolled 54 of these individuals from the COFLU Peru study into Neuro COFLU. Thirty-two were not enrolled given their COFLU Peru study visits were completed before Neuro COFLU start date.

Study participants were invited to participate in Neuro COFLU at visit days 90, 180 or 365 after initial recruitment. A separate additional informed consent was obtained for all participants from the larger study. This Neuro COFLU sub-study was approved by the institutional review board at Universidad Peruana Cayetano Heredia (203010) in Lima, Peru.

### Data collection and analysis

Home visits for each participant took place between January 2022 and December 2022 at days 90, 180, 270 or 365 post COVID-19 diagnosis. A study physician would arrive at the location at a pre-scheduled time and conduct all study procedures within about an hour. The study procedures for Neuro COFLU included: a questionnaire on sociodemographic characteristics, SARS-CoV-2 infection and vaccination information, social, academic and economic impact of COVID-19, subjective percent recovery from COVID-19 (Likert scale of 1–100 where 100% was how the participant felt pre-COVID-19), neurologic diagnosis history, and current neurologic, cognitive and psychiatric clinical symptoms. For each of the current clinical symptoms, they were then asked whether the symptom started before or after COVID-19. For participants seen at each subsequent neurology visit, participants answered follow-up neurologic symptom questions. All data presented here is from the Neuro COFLU sub-study.

Study data were collected and managed using REDCap electronic data capture tools and exported to Excel. Data were summarized as number of patients (frequency), mean (standard deviation) for normally distributed variables, and median [interquartile range (IQR)] for non-normally distributed variables. We present descriptive statistics. For each neurologic symptom reported at each visit, participants were additionally asked if this symptom also was present “before COVID-19.” Symptoms that were present “before COVID-19” were not included in the prevalence to present “new” neurologic symptoms since COVID-19. “New” neurologic symptoms refer to “new” since COVID-19 and not “new” since previous visit. A participant could have the same “new” symptom at multiple visits indicating persistence of the new symptom. Data on symptom severity was not assessed and therefore exacerbations of symptoms existing before COVID-19 are not addressed here.

A logistic regression analysis was performed to examine the association between the new-onset symptom status following COVID-19 (yes vs. no) at 270 days and six risk factors: age (in years), sex (female vs. male), vaccination status at the time of COVID infection (yes vs. no), history of pre-existing depression, anxiety, or headache disorder (yes vs. no for each). Only the new-onset symptoms with a prevalence >10% were included in the analysis. First, a simple logistic model was applied to each symptom status as the dependent variable and each risk factor as an independent variable. Then, a multiple logistic regression model was constructed to adjust for age and sex. A significance level of 0.15 was used for covariate selection. Odds ratio (OR) and 95% confidence intervals (CI) were reported. Model fit was assessed using the Hosmer–Lemeshow (HL) goodness-of-fit test. The Box–Tidwell test was used to evaluate the linearity of the continuous age variable by testing the interaction between age and log (age). A significance level of 0.05 was used for all association tests, as the analysis was exploratory.

SAS software was used to extract the symptom information from the raw data and calculate the descriptive statistics and conduct logistic regression (20). To present distribution of subjective recovery scores longitudinally, GraphPad Prism version 9.0.0 for Mac, GraphPad Software, San Diego, California USA, www.graphpad.com GraphPad was used.

## Results

All 54 participants were seen at least for one visit, 49 (91%) for two visits, and 35 (65%) for three visits. Most of the information was collected from individuals at 180- and 270-days post-infection. Table 1 shows the breakdown of participants seen per visit after COVID-19.

**Table 1:** Sociodemographic characteristics, impact of COVID-19, and participants seen per visit day post-infection (*N* = 54).

Sociodemographic characteristics	Frequency, *n* (%)
Age, median (range)	33 (18–68)
Female sex, *n* (%)	37 (68.5)
Education level completed	
Primary school or less	4 (9)
Secondary school	9 (17)
Some technical/University education	22 (41)
University degree and more	19 (35)
Employment	
Working at time of first visit	43 (80)
In school at time of first visit	10 (19)
In both school and work	9 (17)
History of working or studying before COVID-19	49 (91)
Impact of COVID-19	
Missed >2 weeks of work/school (*n* = 49)	10 (20)
Lost job or missed school due to COVID-19 illness (*n* = 49)	12 (25)
Played sports/exercised before COVID-19 (*n* = 54)	23 (43)
Stopped sports/exercise due to pandemic (*n* = 23)	8 (35)
Stopped sports/exercise due to COVID-19 illness (*n* = 23)	5 (22)
Lost family member to COVID-19 (*n* = 43)	7 (16)
Participants seen per visit day post-infection	
Day 90	29 (54)
Day 180	42 (78)
Day 270	46 (85)
Day 365	21 (39)

Only two of the 54 participants required hospitalization during their SARS-CoV-2 infection. The median age of the participants was 33 years with a range of 18–68 and 37 (68.5%) were female. Regarding education, over three-fourths of our cohort had graduated from secondary school and over a quarter had graduated from university. At the first visit, 43 reported being employed, 10 reported attending school, and 9 both worked and attended school (Table 1).

Seventeen participants (31%) reported a past diagnosis of depression and 10 (19%) reported anxiety. Three-fourths of the cohort reported a prior history of diagnosis of migraines and/or other headaches, whereas the remaining quarter reported no previous neurologic diagnosis history (Table 2).

**Table 2:** Reported neurologic and psychiatric comorbidities before COVID-19 (*N* = 54).

Neurologic/psychiatric comorbidities	Frequency, *n* (%)
Depression	17 (31)
Anxiety	10 (19)
Migraine	13 (24)
Other headache disorder	32 (59)
Seizures	3 (6)
Learning disorder	1 (2)
ADHD	1 (2)
NCC	1 (2)
HIV	1 (2)
No premorbid disorders	13 (24)

ADHD, attention deficit/hyperactivity disorder; NCC, neurocysticercosis.

Seven participants (16%) lost a direct family member to COVID-19. By the first neurologic follow-up visit all participants had received two doses of the COVID-19 vaccine. Thirty-four of the 46 who reported a date of vaccination, reported vaccination prior to presenting with symptoms of COVID-19. Of the 46 that responded regarding vaccine type, over half received Sinopharm followed by a quarter with Pfizer. Among those who worked or studied before the COVID-19 pandemic, 10 (20%) reportedly missed more than 2 weeks of work or school due to their illness and 12 (25%) reported losing their job or dropping out of school due to COVID-19. Twenty-three participants (43%) played sports or exercised regularly before getting sick with COVID-19. Of these participants, 8 (35%) reported that they stopped playing sports or exercising after the pandemic began. Five (22%) participants stopped playing sports or exercising directly related to their illness (Table 2).

### Outcome data

#### Subjective symptom reporting

Table 3 presents the prevalence of each symptom based on day of visit post-COVID-19. A large majority of patients reported at least one new neurologic or neurocognitive symptom since COVID-19, at each follow-up visit with the following breakdown: 24/29 (83%) at 90 days post infection, 31/42 (74%) at 180 days, 41/46 (89%) at 270 days, and 20/21 (95%) at 365 days. The median number of new symptoms based on visit day post-infection was between 3 and 4 symptoms at any different visit. At 90 days, the most common new neurologic symptoms reported included: muscular pain, neck stiffness, headache, loss of appetite, numbness, insomnia, fatigue, and weakness between 24 and 28% for any symptom. At 180 days the most common new symptoms reported were: insomnia, weakness, muscular pain, and numbness between 24 and 31% for any symptom. At 270 days, the most common new neurologic symptoms included: changes in hearing, muscular pain, fatigue, joint pain, and insomnia between 28 and 30%. At 365 days, the most common new neurologic symptoms were: changes in hearing, weakness, fatigue, irritability, and memory problems between 29 and 33%.

**Table 3:** New neurologic symptoms since COVID-19 based on visit day post-infection.

Symptom	Visit day post-infection *n* (%)			
	90 (*n* = 29)	180 (*n* = 42)	270 (*n* = 46)	365 (*n* = 21)
Anxiety	5 (17)	3 (7)	5 (11)	4 (19)
Blurry vision	1 (3)	4 (10)	2 (4)	2 (10)
Burning sensation	6 (21)	6 (14)	9 (20)	2 (10)
Change in behavior	3 (10)	5 (12)	5 (11)	3 (14)
Changes in hearing	5 (17)	9 (21)	14 (30)	7 (33)
Depressed	6 (21)	8 (19)	6 (13)	3 (14)
Difficulty concentrating	3 (10)	6 (14)	9 (20)	4 (19)
Dizziness	3 (10)	6 (14)	8 (17)	0 (0)
Double vision	1 (3)	3 (7)	0 (0)	1 (5)
Fatigue	7 (24)	7 (17)	14 (30)	7 (33)
Feeling room is spinning	1 (3)	4 (10)	2 (4)	2 (10)
Hallucination	0 (0)	2 (5)	0 (0)	0 (0)
Headache	7 (24)	8 (19)	11 (24)	5 (24)
Irritability	4 (14)	6 (14)	8 (17)	6 (29)
Joint pain	4 (14)	9 (21)	13 (28)	3 (14)
Loss of appetite	7 (24)	7 (17)	7 (15)	3 (14)
Loss of smell	1 (3)	5 (12)	2 (4)	3 (14)
Loss of taste	0 (0)	4 (10)	5 (11)	2 (10)
Memory problems	5 (17)	8 (19)	10 (22)	6 (29)
Muscular pain	8 (28)	11 (26)	14 (30)	5 (24)
Neck stiffness	8 (28)	8 (19)	8 (17)	4 (19)
Numbness	7 (24)	10 (24)	10 (22)	3 (14)
Rash	2 (7)	3 (7)	5 (11)	3 (14)
Insomnia	7 (24)	13 (31)	13 (28)	5 (24)
Over sleeps	2 (7)	2 (5)	8 (17)	2 (10)
Thoughts of ending life	1 (3)	1 (2)	1 (2)	1 (5)
Tingling skin	5 (17)	9 (21)	7 (15)	3 (14)
Weakness	7 (24)	11 (26)	11 (24)	7 (33)
Reported at least one neurologic symptom	24 (83)	31 (74)	41 (89)	20 (95)

Certain symptoms, such as headache post-COVID-19 were experienced in a quarter of the cohort at 90, 270, and 365 days. Other symptoms such as memory problems, difficulty concentrating, and fatigue increased steadily in frequency over the course of the year. Change in hearing also increased over the course of the year and more than doubled in frequency in that time. In contrast, the frequency of loss of appetite and numbness steadily decreased over the course of the year.

#### Neurologic symptom associations

At the 270-day visit, the simple logistic analysis found that pre-existing anxiety was significantly associated with the development of new onset hearing changes (OR = 7.25, 95% CI 1.48–35.61, *p* = 0.0147), muscular pain (OR = 15.00, 95% CI: 2.55–88.40, *p* = 0.0028), numbness (OR = 8.00, 95% CI: 1.59–40.33, *p* = 0.0118), and weakness (OR = 6.46, 95% CI: 1.33–31.32, *p* = 0.0206) following COVID-19. Pre-existing depression was associated with new neck stiffness (OR = 8.40, 95% CI: 1.45–48.61, *p* = 0.0175) and numbness (OR = 14.00, 95% CI: 2.46–79.55, *p* = 0.0029) following COVID-19. Age was only weakly (OR = 0.94, 95% CI: 0.88–1.00, *p* = 0.478) associated with new-onset headache following COVID-19 and could be considered as no association with any new onset symptoms. Sex, vaccination status at the time of infection, and pre-existing headache disorder were not significantly associated with any new neurologic symptom after COVID-19 (Table 4).

**Table 4:** Neurologic symptom associations.

Symptom	Risk factor	Simple logistic regression				Multiple logistic regression			
		Odds ratio (OR)	95% CI		*p*-value	Odds ratio (OR)	95% CI		*p*-value
			LowerCL	UpperCL			LowerCL	UpperCL	
Headache	Age	0.94	0.88	1.00	0.05				
Neck stiffness	Pre-depression	8.40	1.45	48.61	**0.02**	7.07	1.14	43.80	**0.0357**
Numbness	Pre-depression	14.00	2.46	79.55	**0.00**	11.85	1.99	70.54	**0.0066**
Hearing changes	Pre-anxiety	7.25	1.48	35.61	**0.01**	**8.52**	**1.36**	**53.30**	**0.0219**
Muscular pain	Pre-anxiety	15.00	2.55	88.40	**0.00**	10.40	1.61	67.14	**0.0139**
Numbness	Pre-anxiety	8.00	1.59	40.33	**0.01**	11.21	1.50	84.14	**0.0187**
Weakness	Pre-anxiety	6.46	1.33	31.32	**0.02**	5.85	1.02	33.49	0.0473

The table represents simple and multiple logistic regression models for the 270-day visit looking at impact of 6 risk factors (age, sex, vaccination status at the time of COVID-19, pre-existing headaches, pre-existing depression, and pre-existing anxiety) on presence of post-COVID-19 new neurologic symptoms. A total of 22 neurologic symptoms had a prevalence >10% and were included in the logistic regression analysis, resulting in a total of 132 tests (22 symptoms × 6 risk factors) in the simple logistic regression and 88 tests (22 symptoms × 4 predictors) in the multiple logistic regression. The multiple regression model included age and sex as covariates. Age and sex were not found to be significant. Odds ratios and 95% confidence intervals are presented along with *p*-values for each of the significant associations. Bolded values represent those of statistical significance.

Most odds ratios were >1 (except for age), indicating that individuals with a pre-existing condition were more likely to develop new-onset symptoms following COVID-19 than those without such a condition. The significant associations identified in the multiple logistic regression model were consistent with those from the simple one, as age and sex were not retained as covariates in the final models (*p* > 0.15). These findings should be interpreted as exploratory, as *p*-values were not adjusted for multiple comparisons.

#### Subjective percent recovery

**Figure 1:**
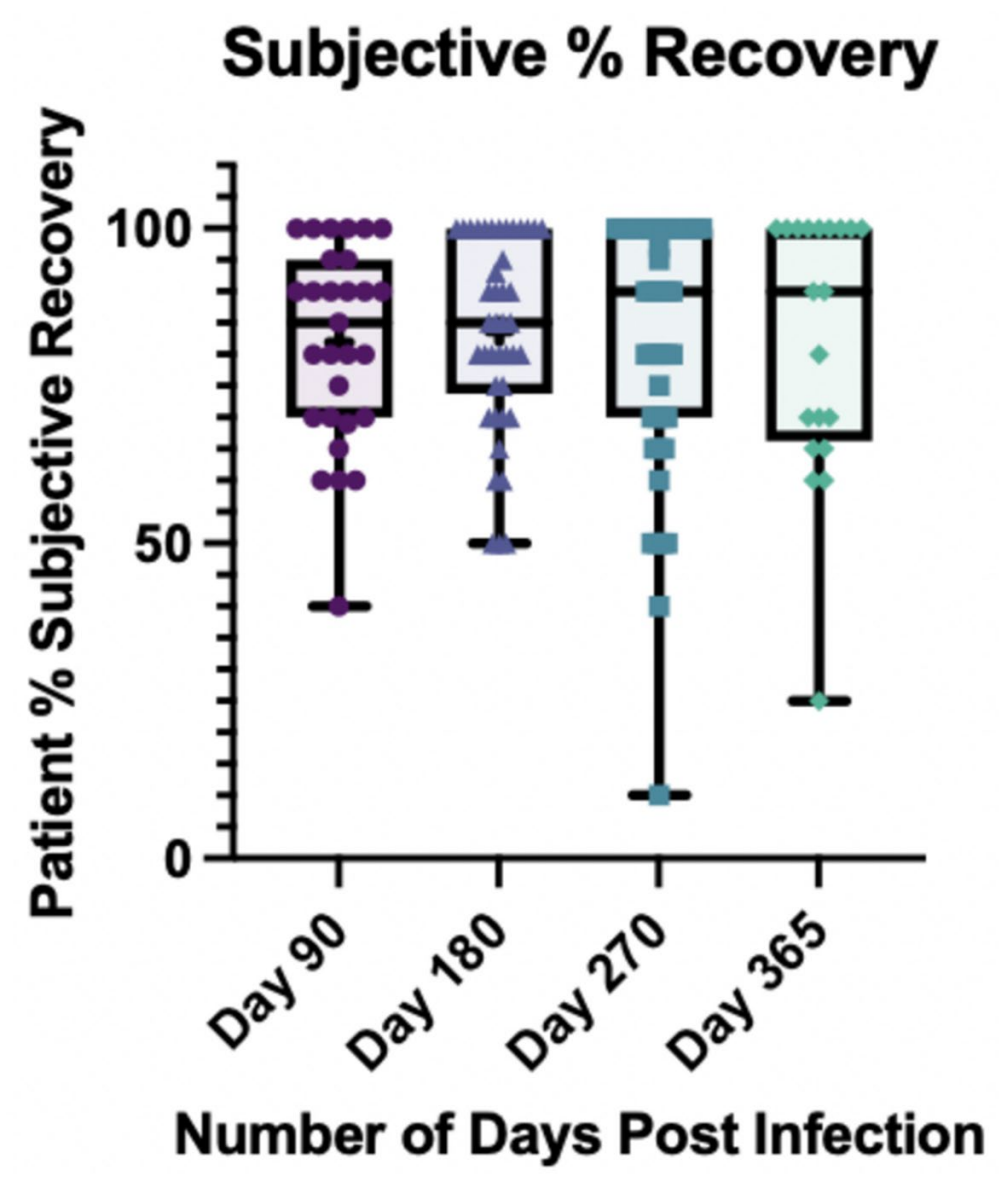
Subjective percent recovery by visit day post-infection (*N* = 53). Participants were asked to rate their percent recovery after COVID-19 from 1 to 100 where 100% represented how they felt pre-infection. *At day 365, one participant had missing data for percent recovery.

Patients were asked to rate their overall subjective percent recovery on a 1–100 Likert scale at each visit where 100% was the baseline of how they felt before becoming ill with COVID-19. Figure 1 demonstrates the distribution of self-reported recovery percentages based on visit date post-COVID-19. The median self-reported percent recovery out of 100 that participants reported at each visit date was 85% at day 90, 85% at day 180, 90% at day 270 and 90% at day 365. At day 90, 6 out of 29 (21%) reported being back to 100% vs. 13 out of 42 (31%) at day 180, 17 out of 46 (37%), and 9 out of 20 (45%) at day 365.

## Discussion

Through this prospective longitudinal observation study of Peruvians with mild to moderate COVID-19 enrolled at symptom onset with confirmatory PCR test in a public hospital emergency room, we found that neurologic symptoms that were new since COVID-19 existed in over three-fourths of participants between 3 months and 1 year post COVID-19. Eighty-three percent of participants reported at least one neurologic symptom at 3 months post-infection which increased to 95% by 1 year. Almost half of the neurologic symptoms increased in frequency within our cohort over the course of the year. The most common symptoms in the first 6 months post-infection were physical signs, such as muscular pain, neck stiffness and insomnia whereas in the second 6 months, more interoceptive symptoms were observed such as fatigue and weakness. Interestingly, at visits in the last 6 months of the year, the most prevalent neurologic symptom reported was new hearing loss. Despite the high burden of neurologic symptoms in this population which increased over time after COVID-19, participants still reported a subjective feeling of recovery with an upward trajectory from 85 to 90% where 100% was their pre-COVID-19 baseline. There was no association of age, sex, or vaccination status at time of infection with post-COVID-19 neurologic symptom presence. It was lastly found that pre-existing headaches had no association with post-COVID-19 neurologic symptoms, however pre-existing anxiety and depression did have a significant association with several symptoms.

Prior to the pandemic, it was estimated that neurological disorders produced more than 10% of disability adjusted life years in Peruvian adults with migraine being the leading cause of neurological burden (21). Our cohort had an almost three fourths prevalence of headaches which is consistent with estimates of 1 year prevalence rates among Peruvian adults of 64.6% prior to the pandemic (22). This suggests generalizability from our cohort to the general population. The prevalence, however, of pre-existing depression and anxiety in our population (31 and 19% respectively) is greater than pre-pandemic estimates which include 6.3% for depression (23) and 14.9% for anxiety (24). Pre-existing anxiety and depression in the present study, though, had significant association with sensory and pain symptoms including numbness, changes in hearing, muscular pain and neck stiffness after COVID-19.

The high burden of neurologic symptoms post-COVID-19 in our cohort are consistent with several other studies completed in high income countries (25–27). One study in the United States which enrolled 50 PCR-positive patients referred to a neuro-COVID-19 clinic at an average of 4.7 months after COVID-19 symptom onset found an overall median of five neurologic symptoms per patient. This compares to a median number of symptoms in our cohort of 3 at between 90 and 180 days. The most frequent symptoms seen in this US cohort included: “brain fog” (81%), headache (68%), numbness/tingling (60%), dysgeusia (59%), anosmia (55%), and myalgia (55%). This group’s subjective recovery score compared to pre-COVID-19 was 67.8% (28). The same group re-assessed the cohort and found no significant change in most neurologic symptoms between the first and second visit at a median of 14.8 months after symptom onset (29).

Peru had a devastating loss of life from COVID-19, and because of challenges to the health care system and scarcity of resources, many patients with severe COVID-19 died in Peru who might have survived in other settings. Thus, our study population was predominantly composed of those who had suffered from mild to moderate disease. Our population was largely non-hospitalized with only 2 participants admitted briefly to a hospital. We found here that despite the mild to moderate acute infection, neurologic symptoms were prevalent at follow-up visits. A similar study conducted in Colombia found not only the persistence of many neurologic symptoms several months after infection, but that both non-hospitalized and hospitalized patients had persistent neurologic symptoms with “numbness or tingling” (58 vs. 24%, *p* < 0.001) and “abnormal body sensations” (42 vs. 12%, *p* < 0.001) being the only symptoms reported significantly more frequently in the hospitalized group (30).

Studies from Peru of neurologic aspects of acute and post-acute COVID-19 are limited. One study on patients with mild to moderate severity COVID-19 in Lima, Peru found that 83% presented with at least one neurologic symptom in the acute stage of infection with headaches and smell/taste dysfunction most commonly reported (31). One study on Peruvian adults in the post-acute period highlighted the cognitive impact of COVID-19 even following mild cases (32). Another multinational study on wellbeing and cognition in older adults found that Peruvians had rated high self-impact of the pandemic especially in the areas of economic, physical distancing and infection history and this correlated with greater perceived discrimination and memory concerns (33).

Our study also provides insight into the health, economic, and educational impact of COVID-19 on Peruvians residing in urban Lima. A quarter of participants who previously had employment or student status lost these opportunities during the pandemic. About 70% of Peru’s workforce is composed of informal workers resulting in severe financial challenges and little public protection during lockdown (12). Given Peru had some of the strictest lockdown policies (12, 34), over a quarter reported stopping all exercise and recreational activities during the pandemic. A study on the cognitive and neuropsychological impact of the COVID-19 mandatory lockdown on Peruvian Alzheimer’s patients found that cognition and neuropsychiatric symptoms worsened in lockdown however neuropsychiatric symptoms demonstrated stability or improvement after lockdown highlighting the great level of stress caused by confinement in Peru (35). Additionally, often family members act as informal caregivers for those with chronic illnesses, and therefore the burden of neurologic impairment spreads beyond the individual afflicted. One study on dementia care found perceived caregiver burden and patient behavioral symptoms were worse during the COVID-19 pandemic in Lima, Peru (36). All the above pose unique additional stressors on participants that may impact symptom experience in the post-COVID-19 recovery period.

Interestingly, there is an apparent discrepancy in the lived experience following COVID-19 in our cohort and their self-reported percent recovery post-COVID-19. Ninety-one percent reported at least one neurologic symptom at day 270, however 37% reported feeling back to 100 and 95% reported at least one neurologic symptom still at day 365 despite 45% reporting return to pre-COVID-19 baseline. This poses the question of how we define recovery from COVID-19. Such populations may not have come forward as having self-identified “Long-COVID” but here are clearly reporting numerous persistent neurologic symptoms that are new since COVID-19 and potentially impacting their return to work and daily life. How the symptoms are perceived post-COVID-19 by the population surveyed may affect the data that is collected and therefore emphasizes further the need to perform such studies in differing contexts. For example, headache syndromes in the general population are less frequent throughout Asia compared to Latin America (37) which may be due to cultural perception of pain or symptom reporting.

A limitation of this study is the lack of a control group. Given the complexity of the pandemic in a low resource setting where PCR test kits to confirm infection were limited and very difficult to find outside of research setting in Peru, it would have been challenging to enroll a population that was definitively never infected with SARS-CoV-2. Antibody surveys demonstrated that more than 75% of adult Americans had evidence of SARS-CoV-2 infection by the end of 2022, and the CDC estimated that 80–90% were infected at least once by the end of 2023 (38). As such, there is limited ability to provide a control group for COVID-19 studies. Even if we had attempted to follow up the patients who were originally SARS-CoV-2 negative at the start of the study, most would have become infected during the subsequent follow up period, making their data invalid.

Additionally, our study was reliant on the self-report of participants regarding symptoms. In an effort to highlight only symptoms possibly attributable to COVID-19, for each symptom reported, we then asked if the symptom was also present before COVID-19. In the case that it was reported before COVID-19, this symptom was not included in our data presented here. This method is unfortunately susceptible to recall bias. In the case of headache, we asked separately in the first visit questionnaire whether participants had a history of diagnosis of headache disorder, of which 24% reported migraine diagnosis and 59% with other headache types. We then asked at each follow up visit in the symptom questionnaire if headache was present and subtracted those that reported having headaches before. About a quarter reported new headaches since COVID-19. As such, it is possible that either those who reported new headaches forgot they had a previous headache diagnosis or considered the headache disorder resolved in the time period prior to COVID-19. Additionally, we did not ask about symptom severity at each visit. We therefore were unable to assess whether there was a change in symptom character over time nor could we address whether pre-existing neurologic symptoms were exacerbated after COVID-19.

To illuminate any predictors of post-COVID-19 neurologic symptoms, we conducted 132 logistic regression analyses. This returned only six significant association between pre-existing anxiety and depression and post-COVID-19 neurologic symptoms. Numbness was associated with both which may be tied to paresthesia associated to hyperventilation in setting of increased anxiety, and the reason for this being new may be heightened anxiety following the pandemic. All the symptoms, except changes in hearing, may represent a physical sign of depression or anxiety. Alternatively, given the large number of analyses done, this result may be a type I error and therefore due to chance.

Our study had a significant amount of loss to follow-up for multiple reasons. Contact with participants was maintained via cell phone contact. It is common for individuals to change phone numbers frequently which contributed to loss of contact. Additionally, many participants had large life changes in the year of the study ranging from geographical moves to hospitalization and loss of loved ones, all of which complicated their ability to participate. The study size was small secondary to attrition from the parent study as well as due to the time frame and budget of the study.

One strength of this Long-COVID neurologic symptom study is that participants were recruited prospectively when having COVID-19 symptoms in the acute setting with the diagnosis confirmed by PCR and were followed longitudinally for development of neurologic symptoms post-acutely. Recruitment for this study did not depend on the existence of neurologic symptoms in the acute or post-acute setting. Many other studies instead analyze neurologic symptoms in those referred for Long-COVID thereby risking potential sample bias. As such, it is especially concerning that so many of our participants report persistent neurologic symptoms. Given our population was recruited from a public hospital in an urban low resource district of Lima, many participants likely have low health care access and may not have access to support for dealing with Long-COVID symptoms. Our study, though small, may represent many unknown sufferers of neurologic Long-COVID. It is important to recognize that it is not just those that present to neurology clinics and long-COVID clinics in high resource settings that have the condition, and that those in under-resourced settings with poor access to health care may be underrepresented in Long-COVID lived experience as well (39). A meta-analysis of acute COVID-19 symptoms and demographics of patients in Latin America demonstrated important differences based on country highlighting that country-specific management and prevention plans are needed (40). A similar consideration should be made for post-acute sequelae following COVID-19.

Given how greatly COVID-19 has impacted low resource settings, especially those in Latin America, it is essential to develop public programs for screening for neurologic, cognitive and psychiatric disability from Long-COVID and strategies for counteracting these post-acute symptoms. Cognitive rehabilitation has been used in some institutions with promising outcomes (41) and efforts should be made to provide similar culturally appropriate resources for Peruvians. This small study emphasizes the potential social and economic impact that Long-COVID has in underrepresented regions and therefore it should be a funding priority to focus resources to rehabilitate and support this population burdened with lasting neurologic sequelae of COVID-19.

## Conclusion

In summary, by following a cohort of patients in the general population of Lima, Peru who had mild to moderate acute COVID-19 symptoms up to one-year post-infection, we found a high burden of neurologic symptoms, many of which persisted many months after their initial identification. Given the impact on social, economic and academic factors in our small population in the aftermath of COVID-19, it is likely this impact is amplified at the national level. Larger studies are needed to further characterize neurologic Long-COVID in different regions globally so that context-specific rehabilitation plans can be developed that help individuals without further increasing health disparities.

## Data Availability

The raw data supporting the conclusions of this article will be made available by the authors, without undue reservation.
