# Correction: Neurologic symptoms following COVID-19 in Lima, Peru: a prospective longitudinal observational study

**DOI:** 10.3389/fneur.2025.1676667

**Published:** 2025-10-10

**Authors:** Hanalise V. Huff, Carla Villanueva-Colina, Monica M. Diaz, Sofia Tovar, Andrea Davila Luna, Tianxia Wu, Davidson H. Hamer, Igor J. Koralnik, Tom Solomon, Miguela A. Caniza, Patricia J. Garcia

**Affiliations:** ^1^Department of Global Health and Population, Harvard T. H. Chan School of Public Health, Boston, MA, United States; ^2^Section of Infections of the Nervous System, National Institute of Neurological Disorders and Stroke, National Institutes of Health, Bethesda, MD, United States; ^3^School of Public Health, Cayetano Heredia University, Lima, Peru; ^4^Department of Neurology, University of North Carolina at Chapel Hill School of Medicine, Chapel Hill, NC, United States; ^5^Clinical Trials Unit, National Institute of Neurological Disorders and Stroke, National Institutes of Health, Bethesda, MD, United States; ^6^Department of Global Health, Boston University School of Public Health, Boston, MA, United States; ^7^Section of Infectious Diseases, Boston University Chobanian and Avedisian School of Medicine, Boston, MA, United States; ^8^Boston University Center on Emerging Infectious Diseases, Boston, MA, United States; ^9^Ken and Ruth Davee Department of Neurology, Northwestern University Feinberg School of Medicine, Chicago, IL, United States; ^10^The Pandemic Institute and The National Institute for Health and Care Research (NIHR) Health Protection Research Unit in Emerging and Zoonotic Infections, University of Liverpool, Liverpool, United Kingdom; ^11^The Walton Centre, NHS Foundation Trust, Liverpool, United Kingdom; ^12^Department of Global Pediatric Medicine and Infectious Diseases, St. Jude Children's Research Hospital, Memphis, TN, United States

**Keywords:** Long-COVID, neurologic, global health, Latin America, COVID-19, Peru, neuroinfectious disease

Affiliation “Ken and Ruth Davee Department of Neurology, Northwestern University Feinberg School of Medicine, Chicago, IL, United States” was omitted for author Igor J. Koralnik. This affiliation has now been added for author Igor J. Koralnik.

Affiliation “School of Public Health, Cayetano Heredia University, Lima, Peru” was omitted for authors Carla Villanueva-Colina, Sofia Tovar, and Andrea Davila Luna. This affiliation has now been added for author Carla Villanueva-Colina, Sofia Tovar, and Andrea Davila Luna.

Author Davidson H. Hamer was erroneously assigned to affiliation “Ken and Ruth Davee Department of Neurology, Northwestern University Feinberg School of Medicine, Chicago, IL, United States.” This affiliation has now been removed for author Davidson H. Hamer.

Author Igor J. Koralnik was erroneously assigned to affiliation “The Pandemic Institute and The National Institute for Health and Care Research (NIHR) Health Protection Research Unit in Emerging and Zoonotic Infections, University of Liverpool, Liverpool, United Kingdom.” This affiliation has now been removed for author Igor J. Koralnik.

This affiliation was a duplication of one of Tom Solomon's affiliation [affiliation 9 and 10 are the same] and erroneously assigned to Igor Koralnik.

Author Carla Villanueva-Colina, Sofia Tovar, and Andrea Davila Luna was erroneously assigned to affiliation “School of Medicine, Cayetano Heredia University, Lima, Peru.” This affiliation has now been removed for authors Carla Villanueva-Colina, Sofia Tovar, and Andrea Davila Luna.

Affiliation “Department of Global Health, Boston University School of Public Health, Boston, MA, United States” was omitted from the paper. This affiliation has now been added for author(s) Davidson H. Hamer.

There was a mistake in Table 1 as published. There is no need for “*n* (%)” to be next to: “education level completed,” or any of the “employment” options, or next to “impact of COVID-19” or the “(n)” next to “participant seen per visit day post-infection” since there is an “*n* (%)” now as a header at the top of the right column. The corrected Table 1 appears below.

**Table 1:** 

**Sociodemographic characteristic**	**Frequency, *n (%)***
Age, *median (range)*	33 (18–68)
Female sex, *n* (%)	37 (68.5)
**Education level completed**	
Primary school or less	4 (9)
Secondary school	9 (17)
Some technical/University education	22 (41)
University degree and more	19 (35)
**Employment**	
Working at time of first visit	43 (80)
In school at time of first visit	10 (19)
In both school and work	9 (17)
History of working or studying before COVID-19	49 (91)
**Impact of COVID-19**	
Missed >2 weeks of work/school (*n =* 49)	10 (20)
Lost job or missed school due to COVID-19 illness (*n =* 49)	12 (25)
Played sports/exercised before COVID-19 (*n =* 54)	23 (43)
Stopped sports/exercise due to pandemic (*n =* 23)	8 (35)
Stopped sports/exercise due to COVID-19 illness (*n =* 23)	5 (22)
Lost family member to COVID-19 (*n =* 43)	7 (16)
**Participants seen per visit day post-infection**	
Day 90	29 (54)
Day 180	42 (78)
Day 270	46 (85)
Day 365	21 (39)

There was a mistake in Table 2 as published. There is no need for “*n* (%)” to be next to the heading “Neurologic/psychiatric comorbidities” in the left column since it is present as part of the heading of the right column. The corrected Table 2 appears below.

**Table 2:** 

**Neurologic/psychiatric comorbidities**	**Frequency, *n (%)***
Depression	17 (31)
Anxiety	10 (19)
Migraine	13 (24)
Other headache disorder	32 (59)
Seizures	3 (6)
Learning disorder	1 (2)
ADHD	1 (2)
NCC	1 (2)
HIV	1 (2)
No premorbid disorders	13 (24)

The original version of this article has been updated.

